# Changing our training paradigms in general surgery residency during the covid-19 outbreak. Short communication

**DOI:** 10.1016/j.amsu.2020.08.049

**Published:** 2020-09-09

**Authors:** Gustavo Mastroianni, Virginia M. Cano Busnelli, Martin de Santibañes, Pablo E. Huespe, Agustin Dietrich, Axel Beskow, Juan Pekolj

**Affiliations:** a Residents for General Surgery, Hospital Italiano de Buenos Aires, Argentina; b Upper GI and Bariatric Surgery, General Surgery Service, Hospital Italiano de Buenos Aires, Argentina; c HPB and Liver Transplant, General Surgery Service, Hospital Italiano de Buenos Aires, Argentina; d Minimally Invasive Surgery, General Surgery Service, Hospital Italiano de Buenos Aires, Argentina; e Thoracic Surgery and Lung Transplant, General Surgery Service, Hospital Italiano de Buenos Aires, Argentina; f Upper GI and Bariatric Surgery Unit, Hospital Italiano de Buenos Aires, Argentina; g General Surgery, Liver Transplant Unit, Hospital Italiano de Buenos Aires, Argentina

## 1 Introduction

The latest threat to global health is represented by the outbreak of the respiratory disease CoVID-19. The epidemiological features and the high exposure of HCW to patients favour the asymptomatic SARS-CoV-2 transmission and residents are not exempt from that risk [1].

Under these considerations we adapted all General Surgery Service (GSS) activities, including the surgical training program and patient care system, to protect our personnel and guarantee full-time working force for the worst scenario. All elective operations were postponed immediately and educational activities such as clinical rounds, plenary lessons and face-to-face activities were suppressed.

To date, there is controverted scientific evidence about the strategies to solve educational demands of our general surgery training programs in the midst of this sanitary emergency.

In this communication, we aim to describe the strategies and changes we introduced in our general surgery residency program during the CoVID-19 outbreak, prioritizing the security of the human resources and maintaining a quality of supervised continuous medical teaching.

## 2 Methods and measures

*Surgical training* *program* *characteristics and* *modifications*

Our GSS performs more than 7000 surgeries per year including liver, pancreas, small bowel and lung transplants. The surgical training program includes 48 residents and 14 fellows. The changes described in this communication were introduced when we shifted to face 2 of pandemic status [2].

### 2.1 Strategies to ensure residents safety and wellbeing

#### 2.1.1 Clear and frequent information

In response to misinformation, a specific channel between the hospital crisis committee and residents was created. A daily status report containing clear and concrete information about the number of CoVID-19 cases, deaths and number of HCW affected was shared with all the institution personnel.

#### 2.1.2 Psychological wellbeing

Firstly, the institution provided a direct line of communication with psychologists and support groups [3]. Secondly, within our service, one-on-one mentorship between residents and faculty were continued through virtual meetings. Also, three questionnaires were designed to identify staff members, residents or fellows that may be experiencing difficulties.

#### 2.1.3 Education on personal protective equipment and CoVID-19 management

**Fig. 1:**
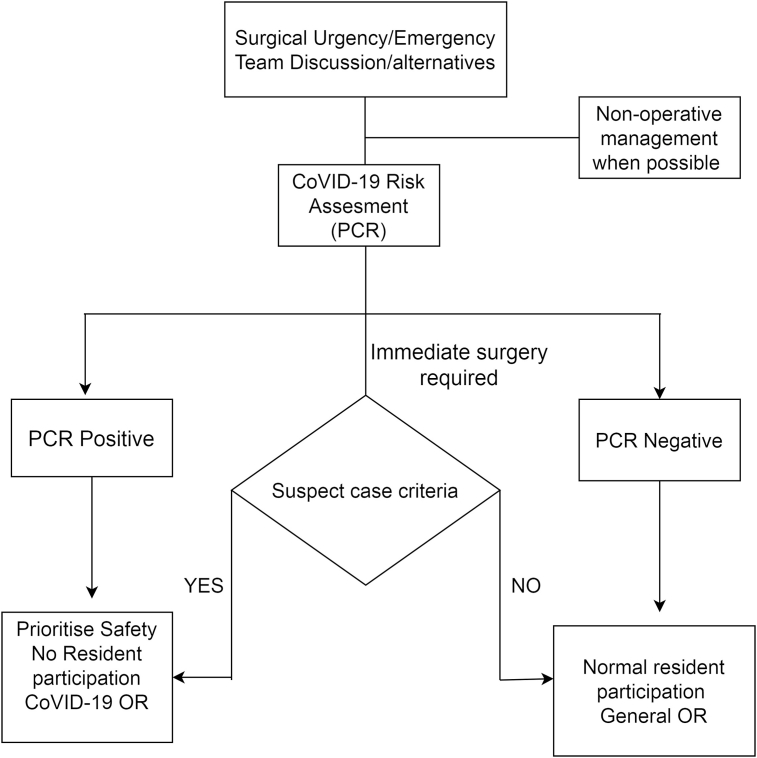
Safety Algorithm for Resident exposure to surgical procedures.

Guidelines for correct use of PPE were created, showing the adequate technique of gowning and doffing [4]. A Safety Algorithm was created to decide Residents participation in OR activity (Fig. 1).

#### 2.1.4 Workforce group-based model tailored to CoVID-19

The SARS-CoV-2 incubation period is about 5 days and most patients who develop symptoms do it within 14 days [1]. Considering this, a one week-on, two week-off model was made. Three groups of residents were established. Two groups attend the emergency department activities in 12-h night and day shifts. A third group is in charge of inpatients and remaining elective procedures. Each group has a senior resident as team leader. Thereby, the amount of health workers required to maintain the GSS is reduced allowing for a “reserve work force”.

### 2.2 Strategies for surgical training enhancement

#### 2.2.1 Non-healthcare assistance activities

Several academic activities were put in place taking advantage of the connecting platforms.

What once were lectures provided by staff members are now classes prepared and conducted by a resident, under online direct supervision of a senior surgeon. Also, every week a senior surgeon shows clinical cases and their solution.

Simulation activities are still in place but were modified to comply with the spacing between residents and the attendance and supervision for the assignments were designated to the housestaff and residents on call for the week.

Furthermore, several CoVID and non-CoVID related research projects experienced acceleration due time availability. To support this, a meeting is carried out once a week between residents and residency program directors in order to guide them with their research projects. The modifications in our training system are portrayed in Table 1.

**Table 1:** Modifications on the residency program in response to the outbreak's consequences.

Program Aspect	CoVID-19 Outbreak Consequences	Response
**Training**	Low exposure to OR activity	Safety algorithm for residents participation in surgeries Teleconferences with Clinical cases and operative videos with tips and tricks Simulation during week-on
	Lack of exposure to emergency situations	12-h shifts one week on- 2 weeks off on emergency department
	Loss of formal teaching sessions and conferences	Scheduled residents-led classes using teleconference tutored by staff
**Research**	Interruption of Research program	Weekly teleconference meetings for follow-up on undergoing projects
	Presenting or authoring research projects	Intensive academic work during week off with tutoring from faculty
**Wellbeing and Safety**	Dealing with unknown entity	Online COVID19 course and daily COVID status reports from the Medical Director
	Safety and use of PPE	Online and hands-on training on PPE usage
	Stress	Open and frequent communication between residents and senior leadership 2 weeks-off allowing for mental rest and relieving the strain on more-than-usual work-related stress

## 3 Results

### 3.1 CoVID-19 cases within residents

A total of 3940 patients with CoVID-19 were admitted in our institution. From a total of 14300 HCW, 312 (2.18%) tested positive for CoVID-19 and one person died. Only one of our staff members had CoVID-19 with mild symptoms (considered community acquired) and up to the date −5 months after the onset of the pandemic in Argentina-no resident contracted the disease.

### 3.2 Workload and residents training

**Fig. 2:**
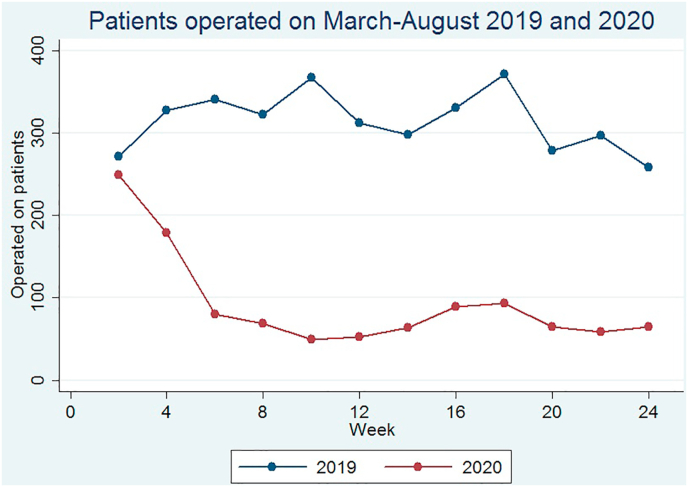
Number of patients operated on march until august 2020 in comparison to the same months of 2019.

**Fig. 3:**
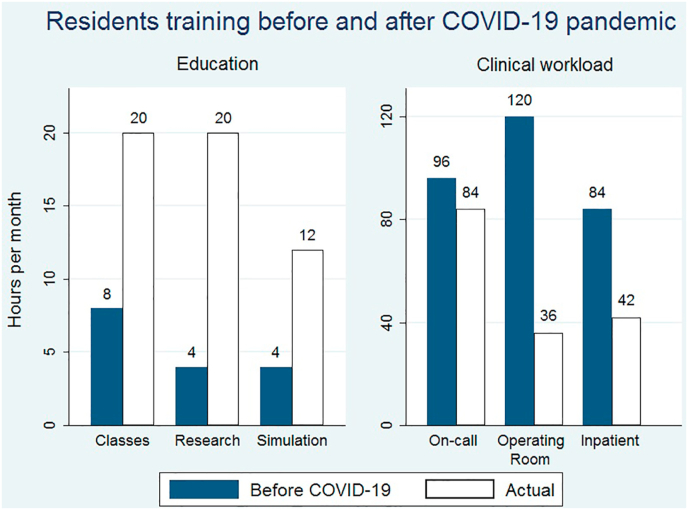
Changes in the hours of training: classes, research activities, simulation and clinical workload spent by the Residents during the pandemic compared to the same period 2019.\

The outpatient clinics were cancelled and a dramatic decrease on surgical procedures was observed (Fig. 2) [5]. The Resident's training program was modified, in order to mitigate the workload reduction, enhancing educational activities and surgical simulation (Fig. 3).

### 3.3 Residents education

Up to date, over 100 classes took place where the residents officiated as lecturers while the staff surgeon in charge was present for comments to the audience. A clinical-based questionnaire was used to assess residents' comprehension of the class. After every class a brief meeting was carried out between the resident and a Staff member. Therefore, feedback was provided about the resident performance. Later evaluation of the answers provided by the residents, as well as a recent satisfaction survey, portrayed that not only residents were able to learn satisfactorily from the classes, but also thoroughly studying for the topic of the class and then lecturing for close to 50 people, gave the residents research-related knowledge, confidence and oratory experience.

### 3.4 Psychological impact and opinion on hospital covid 19 response

Three questionnaires were designed to assess Psychological well-being, resulting in 43% of general surgery personnel experiencing feelings of anxiety with over 50% particularly worried with transmitting the disease to their relatives. 92% of them felt protected and satisfied with the measures implemented in face of the pandemic and 92.5% of the residents felt they were totally satisfied with their training adjustments after the pandemic started.

## 4 Conclusions

We had to learn new ways of teaching in this outbreak onset and take extra measures to assure residents wellbeing.

The group-based model can ensure supervised clinical activities continuity and connecting platforms activities can supply for academic needs. Feedback from the residents is not only welcome but also encouraged and through individual meetings and collective surveys we kept track of the emotions and coping mechanisms that residents were going through. This model proved to be a useful strategy in a general surgery residency program at a university hospital to deal with the outbreak training limitations focusing on resident safety and welfare.

## Funding

This research did not receive any specific grant from funding agencies in the public, commercial, or not-for-profit sectors.

## Provenance and peer review

Not commissioned, externally peer-reviewed.

## Ethical approval

Non required.

## Consent

None required.

## Author contribution

Gustavo Mastroianni, Virginia Cano Busnelli, Pablo Huespe: study design, data analysis and writing.

Martin de Santibañes, Juan Pekolj: study design, writing.

Agustin Dietrich, Axel Beskow: writing.

## Guarantor

Martin de Santibañes.

Juan Pekolj.

## Declaration of competing interest

None.
